# Global insights into energetic and metabolic networks in *Rhodobacter sphaeroides*

**DOI:** 10.1186/1752-0509-7-89

**Published:** 2013-09-13

**Authors:** Saheed Imam, Daniel R Noguera, Timothy J Donohue

**Affiliations:** 1 Program in Cellular and Molecular Biology, University of Wisconsin, Madison, USA; 2 Department of Bacteriology, University of Wisconsin, Madison, Suite 5166, Wisconsin Energy Institute, 1552 University Avenue, Madison, WI 53726-4084, USA; 3 Department of Civil and Environmental Engineering, University of Wisconsin, Madison, USA; 4 DOE Great Lakes Bioenergy Research Center, University of Wisconsin, Madison, USA

**Keywords:** Photosynthesis, Transhydrogenase, Constraint-based analysis, Metabolic modeling, Phenotype microarray, *Rhodobacter sphaeroides*

## Abstract

**Background:**

Improving our understanding of processes at the core of cellular lifestyles can be aided by combining information from genetic analyses, high-throughput experiments and computational predictions.

**Results:**

We combined data and predictions derived from phenotypic, physiological, genetic and computational analyses to dissect the metabolic and energetic networks of the facultative photosynthetic bacterium *Rhodobacter sphaeroides*. We focused our analysis on pathways crucial to the production and recycling of pyridine nucleotides during aerobic respiratory and anaerobic photosynthetic growth in the presence of an organic electron donor. In particular, we assessed the requirement for NADH/NADPH transhydrogenase enzyme, PntAB during respiratory and photosynthetic growth. Using high-throughput phenotype microarrays (PMs), we found that PntAB is essential for photosynthetic growth in the presence of many organic electron donors, particularly those predicted to require its activity to produce NADPH. Utilizing the genome-scale metabolic model iRsp1095, we predicted alternative routes of NADPH synthesis and used gene expression analyses to show that transcripts from a subset of the corresponding genes were conditionally increased in a Δ*pntAB* mutant. We then used a combination of metabolic flux predictions and mutational analysis to identify flux redistribution patterns utilized in the Δ*pntAB* mutant to compensate for the loss of this enzyme. Data generated from metabolic and phenotypic analyses of wild type and mutant cells were used to develop iRsp1140, an expanded genome-scale metabolic reconstruction for *R. sphaeroides* with improved ability to analyze and predict pathways associated with photosynthesis and other metabolic processes.

**Conclusions:**

These analyses increased our understanding of key aspects of the photosynthetic lifestyle, highlighting the added importance of NADPH production under these conditions. It also led to a significant improvement in the predictive capabilities of a metabolic model for the different energetic lifestyles of a facultative organism.

## Background

Information about an organism’s capabilities can be derived from a variety of sources. When genomic information is combined with biochemical, phenotypic or genetic data, functional roles and interrelationships of components within metabolic or regulatory networks become better defined [1-5]. Thus, to obtain a global view of an organism’s capabilities, it is often beneficial to develop models that integrate data from different types of experiments. In obtaining such integrated views, genome-scale metabolic network models can serve both as databases for storage and organization and as tools for the combination and analysis of heterogeneous data sets [6]. A particular interest of our laboratory is developing an integrated understanding of metabolic networks in photosynthetic microbes, because of their abundance in nature, the unique aspects of a solar-driven lifestyle and their growing importance in biotechnological applications [7-9].

We study purple non-sulfur bacteria, a group of photosynthetic microbes that display great metabolic and energetic diversity [10]. The purple non-sulfur bacterium *Rhodobacter sphaeroides* represents one of the best studied photosynthetic organisms, and has been used to develop models of photon capture, light-driven energy metabolism and other aspects of its diverse lifestyles [11,12]. This facultative microbe is capable of anoxygenic photosynthetic growth, aerobic respiration and anaerobic respiration [11,12]. Furthermore, *R. sphaeroides* has been studied for potential biotechnological applications including the ability to produce H_2_[13-15] and ubiquinone [16], production of polyhydroxybutyrate, which can be used as a source of biodegradable plastics [17], remediation of radioactive contamination [18], and its ability to fix CO_2_ and N_2_[7,19,20]. The available genetic, genomic and physiological tools [12] also make *R. sphaeroides* an excellent system in which to improve our understanding of solar energy capture, metabolic and energetic aspects of photosynthesis and other energetic pathways, and the networks which regulate processes of societal and biotechnological interest. To obtain an integrated understanding of photosynthesis or other aspects of *R. sphaeroides*’ lifestyles requires the use of high-throughput data to develop better predictive models of its metabolic network.

In this work, we take a systematic approach to expand our knowledge of the metabolic and energetic networks of *R. sphaeroides* by combining data from genetic, phenotypic and transcriptional analyses with constraint-based modeling. We use high-through phenotypic microarrays to show that wild type *R. sphaeroides* grows on a diverse array of substrates and that this nutrient utilization profile varies significantly between photosynthetic and non-photosynthetic growth conditions. Using the conserved bioenergetic enzyme pyridine nucleotide transhydrogenase (PntAB) as an example, we identify carbon sources where recycling of pyridine nucleotides by this enzyme is essential for photosynthetic or non-photosynthetic growth. We use a genome-scale metabolic model to predict flux distributions and identify alternative NADPH producing reactions that can compensate for the loss of PntAB and thereby explain the conditional growth of *ΔpntAB* cells on selected carbon sources. Transcriptional and phenotypic analyses of defined single and double mutants were used to verify the potential use of some of these alternative NADPH producing reactions under defined conditions. The new data derived from analyzing the growth of wild type and mutant cells were used to develop iRsp1140, a significant update to the existing genome-scale reconstruction of the *R. sphaeroides* metabolic network [11], with increased coverage of metabolic pathways and improved predictive ability. iRsp1140 accounts for 1140 genes, 878 metabolites and 1416 reactions. This work illustrates the new insights into important cellular processes that can be acquired by integrating data from genetic, genomic and other complementary experiments into predictive models of biological systems.

## Results and discussion

### Global analysis of substrate utilization by *R. sphaeroides*

One important step in acquiring a global understanding of cellular processes in an organism is to develop a broad perspective of its metabolic repertoire. An assessment of the literature reveals that *R. sphaeroides* has been reported to grow on 27 carbon, 3 nitrogen, 1 phosphorus and 4 sulfur sources [11]. In contrast, the existing genome-scale model of *R. sphaeroides* metabolic network, iRsp1095, predicted an ability to grow on a significantly larger number of substrates (Table 1) [11]. Thus, to improve our knowledge of the metabolic capabilities of *R. sphaeroides*, and aid subsequent analyses of genetic or physiological perturbations, we used phenotype microarrays (PM) [21,22] to assess the ability of wild type (WT) cells to utilize carbon sources under anoxygenic photoheterotrophic conditions (using a single carbon source as an external electron donor; this is hereafter referred to as photosynthetic growth), aerobic and anaerobic respiratory conditions (see Methods). We also determined the suite of, nitrogen (N), phosphorus (P) and sulfur (S) sources that do or do not support photosynthetic growth.

**Table 1:** **Substrate utilization profile of *R. sphaeroides *under different growth conditions**

**Nutrient source**	Previously known*	Predicted by iRsp1095**	**Based on PM assay**^**a**^		
			**Photo**	**Aero**	**Anaerobic**^**b**^
**Carbon**	27	64	61 (190)	68 (190)	16 (41)
**Nitrogen**	3	31	66 (95)		
**Phosphorus**	1	6	42 (59)		
**Sulfur**	4	4	18 (35)		
**Total**	35	105	187 (379)		

*Previously known growth substrates under photosynthetic conditions.

**Includes 28 false positive predictions and predictions for 20 substrates not included on the various Biolog plates. The simulations for substrate utilization in iRsp1095 were done under aerobic and photosynthetic conditions.

^a^The values in parenthesis represent the total number of carbon, nitrogen, phosphorus or sulfur sources tested using PM and related approaches.

^b^Photo – photosynthetic growth; Aero – aerobic respiratory growth; Anaerobic – anaerobic respiratory growth using DMSO as the terminal electron acceptor.

### R. sphaeroides utilizes different arrays of nutrients across growth conditions

The results from analysis of substrate utilization by WT *R. sphaeroides* cells (Table 1, Additional file 1: Tables S1-S4), significantly expands the array of compounds that support growth of this organism. While the carbon utilization profiles were largely similar during photosynthetic and aerobic respiratory growth, several important differences were observed. Eight carbon sources appeared to support growth photosynthetically but not aerobically, while 15 supported growth aerobically but not photosynthetically (Additional file 2: Figure S1A, Additional file 1: Table S1). Potential causes for the observed differences might include: (i) longer lag times under individual conditions (Additional file 2: Figure S1B), which may result in an apparent inability to utilize the carbon source under one experimental condition; (ii) insurmountable metabolic, bioenergetic or regulatory bottlenecks (Additional file 2: Figure S1C); or (iii) potential differences between the data derived from the photosynthetic PM assay (which measures an increase in optical density) and the aerobic PM assay (that measures respiration) [23].

Of the 53 carbon sources that were used both photosynthetically and aerobically, 41 were tested for their ability to support growth under anaerobic respiratory conditions using dimethyl sulfoxide (DMSO) as the terminal electron acceptor (Additional file 1: Table S1). Only 16 of these carbon sources were capable of supporting anaerobic respiratory growth (as measured by an increase in optical density) after 10 days of incubation. We propose that the inability of WT *R. sphaeroides* to grow in the presence of several carbon substrates during anaerobic respiratory growth is likely due to regulatory and/or bioenergetic constraints, as pathways required for their catabolism are either known or predicted to be present in the genome.

PM assays also revealed that 66 nitrogen, 42 phosphorus and 18 sulfur sources supported growth photosynthetically in WT *R. sphaeroides* (Table 1, Additional file 1: Tables S2-S4). This is a number of nitrogen, phosphorous and sulfur substrates which is similar to those shown to support growth of other well-studied facultative bacteria like *Escherichia coli*[24] and *Bacillus subtilis*[25].

The ability of *R. sphaeroides* to grow on a wide variety of carbon, nitrogen, phosphorus and sulfur sources (Additional file 1: Table S8) is a further demonstration of its metabolic versatility. Of particular interest for future studies is the pattern of substrate utilization observed under different growth conditions, which we propose likely reflects regulatory differences, since enzymes needed to carry out the required reactions are predicted to be encoded in the genome. Below we show that these PM analyses of WT cells provide important reference points for studying the effects of mutations on the metabolic, energetic and regulatory pathways that are potentially used during various modes of growth.

### Analyzing the role of PntAB under defined growth conditions

To illustrate how knowledge of the substrate utilization profile of WT *R. sphaeroides* can be used to assess the effects of genetic perturbation on the metabolic network, we describe insights gained from analyzing an important and widely conserved energetic enzyme, **p**yridine **n**ucleotide **t**ranshydrogenase (PntAB). PntAB is a heterotetrameric membrane-bound enzyme consisting of α and β subunits that catalyzes the reversible exchange of reducing equivalents between pyridine nucleotides based on the magnitude of the proton gradient across the cytoplasmic membrane [26,27].

$$\begin{array}{c} H_{\text{periplasm}}^{+}+\text{NAD}P^{+}+\text{NADH}↔\text{NADPH}+\text{NA}D^{+}\\ \;+H_{\text{cytosol}}^{+} \end{array}$$

Thus PntAB plays a major role in maintaining the balance of cellular pyridine nucleotides (NADH/NADPH). NADPH is a source of reducing equivalents in a large number of crucial anabolic pathways such as the Calvin cycle in autotrophic cells, fatty acid biosynthesis and tetrapyrrole or pigment biosynthesis in photosynthetic organisms [28].

Extensive studies have shown that *E. coli* PntAB expression is induced when there is a demand for NADPH [29]. In addition, *E. coli* PntAB is required for optimal growth on carbon sources whose metabolism does not directly generate NADPH, such as glycerol [29]. *E. coli* also possesses an energy independent soluble transhydrogenase, UdhA, which is induced when there is an excess of NADPH (e.g., growth on acetate) and mediates conversion of NADPH to NADP^+^[29,30]. In addition to PntAB and UdhA, glucose-6-phosphate dehydrogenase (Zwf) and isocitrate dehydrogenase (Icd) can help maintain bacterial NADPH pools under specific conditions [29,30]. We compared growth of *R. sphaeroides* wild type and Δ*pntAB* cells (PntA1 [31]) using PMs to identify conditions with an increased need for NADPH in this bacterium.

### PntAB is conditionally essential for photosynthetic and aerobic respiratory growth

**Figure 1:**
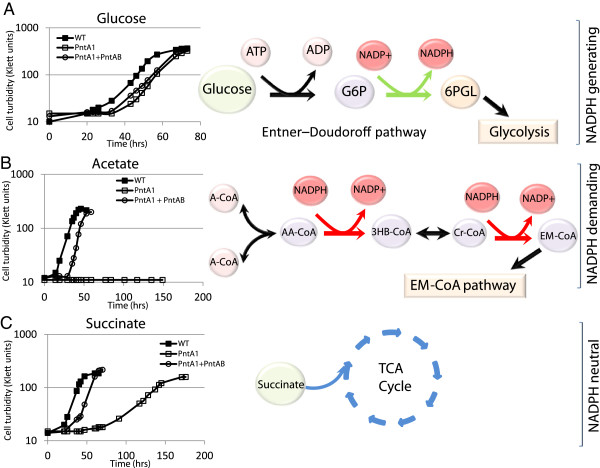
**Categorization of growth substrates based on NADPH demand and requirement for PntAB under photosynthetic conditions. (A) ***R. sphaeroides* growth on glucose via the Entner-Doudoroff pathway includes the NADPH-generating glucose-6-phosphate (G6P) dehydrogenase enzyme (Zwf), which supports normal photosynthetic growth even in the absence of PntAB. **(B)***R. sphaeroides* growth on acetate is predicted to occur via the ethylmalonyl-CoA (EM-CoA) pathway, involving oxidation of 2 molecules of NADPH per 3 molecules of acetyl-CoA (A-CoA) consumed [59]. Note that a third molecule of A-CoA used up in later steps of the EM-CoA pathway is not depicted in this illustration. **(C)** During photosynthetic growth on substrates that do not directly produce NADPH (e.g. succinate), PntAB is required. In some cases, after a long lag period, cells adapt and grow photosynthetically albeit at a slower growth rate (17.2 ± 0.56 hrs compared to 7.53 ± 0.56 hrs for wild type cells). Abbreviations: AA-CoA – acetoacetyl-CoA; 3HB-CoA – 3-hydroxy butyryl-CoA; Cr-CoA – crotonyl-CoA; 6PGL – phosphoglucono-δ-lactone.

Under photosynthetic conditions, only 25 carbon sources supported growth of PntA1 compared to 61 substrates that were used by WT cells. Importantly, only PntA1 cells using D-glucose achieved a final optical density that was equivalent to that of its parent, while PntA1 cells using D-aspartate grew well, whereas the WT parent did not grow on this substrate (Table 2, Additional file 1: Table S5), suggesting these were the only tested carbon sources that supported normal photosynthetic growth in PntA1. To independently confirm the differences observed in the PM assays, we compared photosynthetic growth between PntA1, its WT parent and a PntA1 cells expressing PntAB from an IPTG inducible plasmid (Figure 1). The combined results of these analyses can place the carbon sources that support photosynthetic growth in *R. sphaeroides* into 3 groups (Figure 1A-C, Additional file 1: Table S7). Group I carbon sources such as D-glucose, result in net production of NADPH during their metabolism, via enzymes like the glucose-6-phosphate dehydrogenase. These Group I carbon sources support comparable photosynthetic growth in WT and PntA1 cells (Figure 1A, Additional file 2: Figure S2). Group II carbon sources (such as acetate) are incapable of supporting photosynthetic growth in the absence of PntAB. Metabolism of these Group II carbon sources require net consumption of NADPH, in addition to that required for anabolic processes (Figure 1B, Additional file 2: Figure S2). Growth of PntA1 using Group III carbon sources (such as succinate and many others, Additional file 1: Table S7), was significantly impaired compared to that of WT cells, and exhibited a long lag before growth commenced (Figure 1C). It should be noted however, that several Group III carbon sources such as succinate, failed to support photosynthetic growth on agar plates (Additional file 2: Figure S2), reinforcing the need for PntAB activity when using these carbon sources.

**Table 2:** Summary of carbon utilization in WT and PntA1 cells under aerobic conditions

**Total number of carbon sources utilized**	**Photo**^**a**^	**Aero**
PntA1	25	51
WT	61	73
**Differences in carbon sources utilized**		
Equivalent growth in PntA1 and WT	1	40
PntA1 only	1	1
Increased growth in PntA1	0	1
Reduced growth in PntA1	23	9
Growth in WT only	37	23

^a^Photo – photosynthetic growth; Aero – aerobic respiratory growth.

Combined, these data suggest that PntAB is the major source of NADPH for photosynthetic growth on carbon sources except glucose and D-aspartate. Furthermore, the fact that PntA1, but not its WT parent grows on D-aspartate suggests either that this strain contains unlinked mutations that allow it to metabolize this substrate or that the metabolism of the substrate is induced when the NADPH pool is reduced. This observation could also indicate that *R. sphaeroides* possesses an NADPH-linked pathway for aspartate catabolism [32,33], even though its genome does not contain an open reading frame with significant amino acid sequence similarity to known enzymes with such an activity. In the case of Group III carbon sources (succinate and many others), other NADPH producing pathways could be activated to support growth albeit at a slower rate. For example, the delayed photosynthetic growth that is seen with some Group III carbon sources might be the result of metabolic or genetic alterations that are needed to provide PntAB-independent routes for NADPH synthesis (see below). In addition, we predict that for Group II substrates (such as acetate), the date predict that the combined NADPH demand for metabolism and anabolic processes is too high to be provided by such alternative pathways.

**Figure 2:**
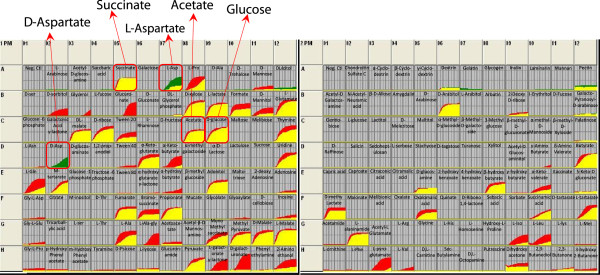
**Growth data from Biolog PM1 and PM2A for WT and PntA1 under aerobic respiratory conditions.** The plots compare the respiration rates across 190 carbon sources between wild type and PntA1 cells. Regions of the graph in red indicate better growth by WT cells than PntA1, while portions in green indicate better growth in PntA1. Regions in yellow represent the overlap between the kinetic data from the 2 strains.

In contrast, PM assays show that under aerobic conditions PntA1 grows similarly to its WT parent on the many of carbon sources assessed (Figure 2, Table 2, Additional file 1: Table S6). Of the 73 carbon sources tested, 40 allowed essentially equivalent growth (as measured by the respiratory output of the PM assays) between PntA1 and its WT parent. In addition, 23 carbon sources that allowed aerobic growth of the WT parent did not support aerobic respiration/growth of PntA1 after 96 hrs of incubation, while another 9 showed significantly reduced aerobic respiration/growth in cells lacking PntAB. Both L- and D-aspartate, supported improved aerobic respiration/growth in PntA1 cells compared to its WT parent (Figure 2). These results underscore potential differences in the relative need for PntAB activity under distinct metabolic states and provide a further indication that an, as yet unidentified NADPH-linked pathway for metabolism of aspartate and potentially other carbon sources exists in *R. sphaeroides*.

### Alternative NADPH-generating pathways can be utilized under different growth conditions

**Figure 3:**
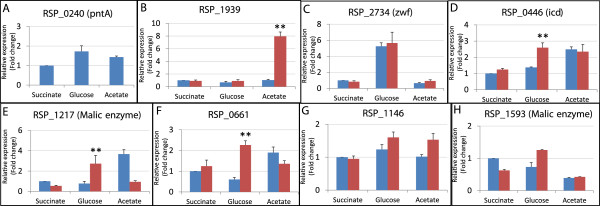
**Transcript levels of genes encoding putative NADPH-generating enzymes in *R. sphaeroides *under aerobic respiratory conditions.** Transcript levels for genes encoding enzymes predicted by iRsp1095 to be involved in NADPH generation (Table 3), were assayed via qRT-PCR in the presence or absence of PntAB. (A) RSP_0240 (pntA) (B) RSP_1939 (NADPH-ferredoxin reductase) (C) RSP_2734 (zwf) (D) RSP_0446 (icd) (E) RSP_1217 (Malic enzyme) (F) RSP_0661 (5,10-methylene-tetrahydrofolate dehydrogenase) (G) RSP_1146 (Glutamate synthase) (H) RSP_1593 (Malic enzyme). Transcript levels in WT cells is shown in blue bars, while those for PntA1 is in red bars. All fold change values represent expression relative to WT succinate-grown cells, whose relative expression was set to 1. ** Significantly increased expression in PntA1 relative to WT.

For Group II and III carbon sources (such as acetate and succinate respectively), which require PntAB for photosynthetic growth, growth of PntA1 was indistinguishable from its WT parent under aerobic respiratory conditions (Figure 2, Additional file 1: Table S6, Additional file 2: Figure S2). One explanation for this observation is that alternative NADPH-generating reactions can function to replace PntAB under aerobic respiratory conditions. Indeed, iRsp1095, predicts 6 other routes that could generate NADPH under defined conditions in *R. sphaeroides* (Table 3). The relative expression of these candidate alternative NADPH-generating pathways was assayed using quantitative reverse transcriptase PCR (qRT-PCR) to determine if transcript levels for the respective genes are increased in the absence of PntAB when compared to a parent strain, when cells were grown by aerobic respiration in cultures containing one of the 3 groups of carbon sources defined above (i.e., glucose, acetate and succinate) (Figure 3A-H). This analysis revealed carbon source-dependent differential expression of potential alternative NADPH generation pathways. During aerobic respiratory growth on a carbon source that requires NADPH consumption (acetate), the loss of PntAB was accompanied by an ~8 fold increase in expression of a putative NADPH:ferredoxin reductase gene (RSP_1939). When using glucose, a carbon source that generates net NADPH, we found that *zwf* (RSP_2734, glucose-6-phosphate dehydrogeanse) transcript levels are increased >5 fold during aerobic respiratory growth on glucose compared to acetate or succinate, in both the WT parental strain and PntA1. Furthermore, transcripts for genes encoding putative malic enzyme (RSP_1217), isocitrate dehydrogenase (RSP_0446) and methylenetetrahydrofolate dehydrogenase (RSP_0661) enzymes were each increased >2 fold in PntA1 during aerobic respiratory growth compared to its WT parent (Figure 3A-H). We recognize that changes in gene expression may not necessarily result in equivalent changes in flux through the corresponding reactions in the direction of NADPH synthesis (see below). However, these data illustrate the potential of alternative NADPH-generating pathways during aerobic respiratory growth to contribute to the growth of PntA1 when using many carbon sources.

**Table 3:** Reactions predicted by iRsp1095 to be involved in NADPH generation

**Gene identifier**	**Enzyme name**	Reaction catalyzed*	**EC number**
RSP_0239 & RSP_0240	PntAB	NADP^+^ + NADH + 2 H^+^[p] = > NADPH + NAD^+^ + 2 H^+^	1.6.1.2
RSP_1939	NADPH-ferredoxin reductase	Reduced ferredoxin + NADP^+^ + H + <= > Oxidized ferredoxin + NADPH	1.18.1.2
RSP_2734	Zwf	D-Glucose 6-phosphate + NADP^+^ < = > 6PGL + NADPH	1.1.1.49
RSP_1217	Malic enzyme	(S)-Malate + NADP^+^ = > Pyruvate + CO_2_ + NADPH	1.1.1.40
RSP_1593			
RSP_0661	THF dehydrogenase	mlthf + NADP^+^ < = > methf + NADPH	1.5.1.5
RSP_0446	Icd	Isocitrate + NADP^+^ < = > 2-Oxoglutarate + CO_2_ + NADPH	1.1.1.42
RSP_1559			
RSP_1146 & RSP_1149	Glutamate synthase	2 L-Glutamate + NADP^+^ < = > L-Glutamine + 2-Oxoglutarate + NADPH + H^+^	1.4.1.13

*6PGL – D-Glucono-1,5-lactone 6-phosphate; mlthf – 5,10-Methylenetetrahydrofolate; methf – 5,10-Methenyltetrahydrofolate; THF dehydrogenase – 5,10-methylene-tetrahydrofolate dehydrogenase.

Interestingly, none of the transcript levels for the tested genes were significantly increased in the succinate-grown PntA1 cells under aerobic respiratory conditions. This could indicate that the contribution of PntAB is minor under this condition and that alternative NADPH-generating pathways provide sufficient NADPH to support growth. Alternatively, other as yet unidentified NADPH-generating pathways or post-transcriptional events might exist in both wild type and *ΔpntAB* cells.

**Figure 4:**
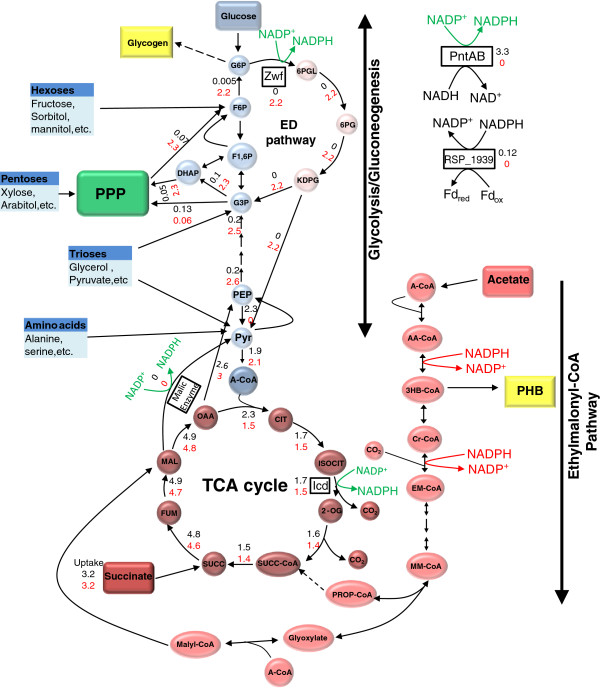
**Predicted flux distributions during aerobic respiratory growth with succinate.** Map of *R. sphaeroides* central carbon metabolism showing flux distributions predicted by iRsp1095 during aerobic growth with succinate. All fluxes are in mmol/gDW h. The fluxes in black are those predicted for the wild type cells, while those in red are predictions made for the Δ*pntAB* deletion strain. Reactions with no flux values are predicted to have a zero flux. To improve accuracy of predictions, fluxes were constrained using publicly available genome-wide expression data from wild type cells grown aerobically on succinate (see Methods). Green arrows indicate the predicted NADPH generating reactions under these conditions. The entry point of some other carbon sources utilized by *R. sphaeroides* are also shown (blue boxes). It should be noted that the predicted fluxes shown represent only one of many optimal solutions from the FBA solution space. Also note that *R. sphaeroides* does not possess a homolog of 6-Phosphogluconate dehydrogenase, which links the pentose phosphate pathway (PPP) with the Entner-Doudoroff (ED) pathway in some other organisms. TCA – tricarboxylic acid; G6P – Glucose 6-phosphate; F6P – Fructose 6-Phosphate; F1,6P – Fructose 1,6-bisphosphate; G3P – Glyceraldehyde 3-phosphate; DHAP – Dihydroxyacetone phosphate; 6PGL – phosphoglucono-δ-lactone; 6PG – 6-Phosphogluconate; KDPG – 2-Keto-3-deoxy-6-phosphogluconate; PEP – Phosphoenolpyruvate; Pyr – Pyruvate; A-CoA – Acetyl-CoA; CIT – Citrate; ISOCIT – Isocitrate; 2-OG – 2-oxoglutarate; SUCC-CoA – Succinyl CoA; SUCC – Succinate; FUM – Fumarate; MAL – Malate; OAA – Oxaloacetate; AA-CoA – acetoacetyl-CoA; 3HB-CoA – 3-hydroxy butyryl-CoA; Cr-CoA – crotonyl-CoA; EM-CoA – Ethylmalonyl-CoA; MM-CoA – β-methylmalyl-CoA; PROP-CoA – Propionyl-CoA; PHB – Polyhydroxybutyrate.

**Figure 5:**
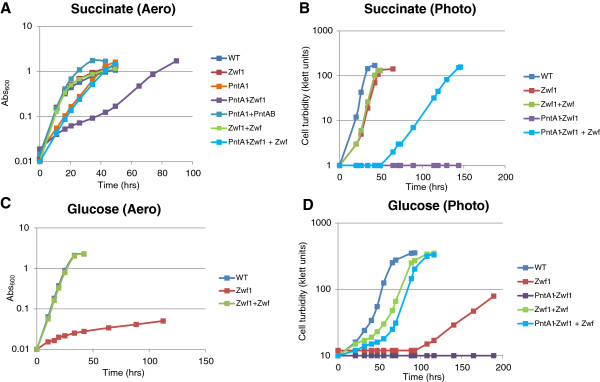
**The role of Zwf during growth with succinate.** Comparison of the growth rates of wild type (WT), PntA1 and PntA1 + PntAB cells, to *zwf* deletion strains Zwf1 and PntA1-Zwf1 and complemented strains Zwf1 + Zwf and PntA1-Zwf + Zwf during aerobic respiratory **(A** and **C)** and photosynthetic **(B** and **D)** growth using succinate or glucose, respectively.

The metabolic flux predictions from iRsp1095 predict that gluconeogenesis is one potential route which could be utilized during growth on succinate in the absence of PntAB, to produce glucose-6-phosphate, which would then be metabolized via the Entner-Doudoroff pathway to produce NADPH (Figure 4). To test this hypothesis, we experimentally analyzed the impact of loss of Zwf under different growth and nutrient conditions. We found that loss of Zwf alone (strain Zwf1) did not significantly impair aerobic respiratory growth on succinate, consistent with the prediction that this enzyme is not required for growth under these conditions. However, loss of Zwf in PntA1 (PntA1-Zwf1) resulted in a significant decrease in aerobic respiratory growth on succinate compared to the PntA1 mutant (Figure 5A), suggesting that Zwf makes a significant contribution to NADPH production, but only in the absence of PntAB, even though *zwf* transcript levels are not increased in PntA1 cells under this condition. Additionally, the observed growth difference between the Zwf1 and PntA1-Zwf1 mutants indicates that PntAB makes even a more significant contribution to NADPH production when cells are grown aerobically with succinate as a carbon source (Figure 5A). These data confirm the model’s prediction and suggest that Zwf is a major NADPH generating enzyme utilized by the cell in the absence of PntAB during aerobic respiratory growth on succinate.

Under photosynthetic conditions, the PntA1-Zwf1 double mutant was incapable of growth on either glucose, succinate or acetate (representatives of each of the 3 major classes of carbon source we defined previously). However, growth is partially restored for glucose and succinate when the PntA-Zwf1 mutant is complemented with a plasmid containing the *zwf* gene (Figure 5B,D). These findings indicate that Zwf also serves as a major route for NADPH generation during photosynthetic growth with these carbon sources, an observation that was predicted by metabolic flux analysis in iRsp1095 (Additional file 2: Figures S3 and S4). Additionally, growth of Zwf1 cells is impaired when using glucose either photosynthetically or aerobically (Figure 5C,D, Additional file 2: Figure S2), suggesting that the Entner-Doudoroff pathway is the major glycolytic pathway in *R. sphaeroides*, despite significant expression of genes encoding enzymes of the Embden-Meyerhof-Parnas pathway in these cultures. This conclusion is also consistent with both experimental analysis of ^13^C-glucose metabolism in *R. sphaeroides* under aerobic respiratory conditions [34] and with the metabolic flux predictions made by iRsp1095 (Additional file 2: Figure S4).

**Figure 6:**
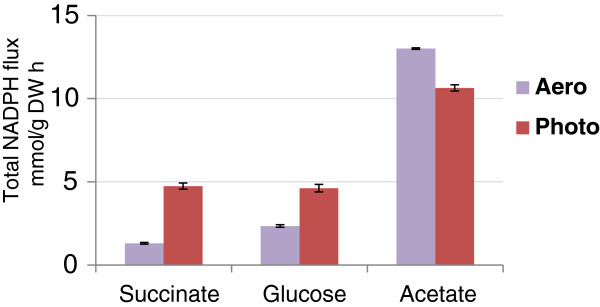
**Comparison of the predicted NADPH flux during photosynthetic or aerobic respiratory growth.** Predictions were made for the total NADPH flux during growth with succinate, glucose and acetate under photosynthetic and aerobic respiratory conditions. The error bars represent the standard error of mean of 1000 fluxes through this reaction obtained from 1000 alternative optimal solutions (see Methods). It should be noted that the predicted larger NADPH demand under aerobic conditions compared to photosynthetic conditions during growth with acetate is due to a larger predicted uptake rate of acetate under aerobic conditions.

The conditional ability of alternative pathways to compensate for the loss of PntAB could reflect a higher demand for NADPH during photosynthetic or other energetic conditions. For example, under photosynthetic conditions, *R. sphaeroides* produces significantly larger amounts of fatty acids, photopigments and other components of the photosynthetic apparatus [35] that are each synthesized via NADPH-dependent pathways. Indeed, iRsp1095 predicts there is a 2–4 fold increase in the demand for NADPH under photosynthetic conditions in cells using succinate or glucose as a major carbon source (Figure 6). If this prediction is accurate, our data suggests that this significantly greater demand for NADPH during photosynthetic growth cannot be met by using alternative NADPH-generating pathways that are sufficient under aerobic respiratory conditions. However, iRsp1095 also predicts that the need for NADPH during aerobic respiratory growth in the presence of acetate is greater than that required for photosynthetic growth on either succinate or glucose, indicating that the cell might have the metabolic capacity to generate sufficient amount NADPH to support photosynthetic growth via these alternative pathways. Thus, additional studies are needed to determine if there are additional constraints under photosynthetic conditions which make alternative NADPH-generating pathways that function under aerobic respiratory conditions insufficient to support growth under other energetic states. Combined, these data illustrate the centrality, and previously unrecognized importance, of the routes for NADPH production in the photosynthetic lifestyle of *R. sphaeroides*. Given the ubiquitous nature of PntAB across biology, it is possible that this enzyme plays a major energetic role in other photosynthetic and non-photosynthetic organisms.

### iRsp1140: a revised experimentally-validated genome-scale metabolic reconstruction for *R. sphaeroides*

The above results provide new information about the metabolic capabilities of *R. sphaeroides* that can be utilized to refine biochemically, genetically and genomically structured databases employed in constraint-based analysis [36]. We previously constructed and validated a genome-scale metabolic reconstruction for *R. sphaeroides*, called iRsp1095, using its annotated genome, published organism-specific data and information from continuous cultures of WT cells [11].

**Figure 7:**
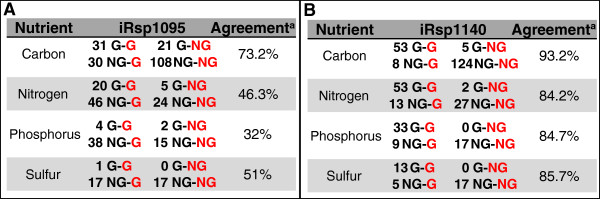
***R. sphaeroides *substrate utilization and transport.** A detailed comparison between the predicted substrate utilization in iRsp1095 **(A)** and iRsp1140 **(B)** with data obtained from PM analysis. G-G – **G**rowth (predicted by model) and **G**rowth (observed in PM); G-NG – **G**rowth (predicted by model) and **N**o **G**rowth (observed in PM); NG-G – **N**o **G**rowth (predicted by model) and **G**rowth (observed in PM); NG-NG – **N**o **G**rowth (predicted by model) and **N**o **G**rowth (observed in PM). ^a^Percentage represents agreement between predictions and PM data from photosynthetic analysis only.

However, the substrate utilization predictions of iRsp1095 could not explain a large amount of the data obtained during PM analysis of wild type and mutant cells (Figure 7A). Consequently, we performed a 2-step refinement and extension of iRsp1095 that involved addition and removal of appropriate reactions, metabolites and genes to increase its agreement with the PM data, while also incorporating newly available metabolic data for *R. sphaeroides* since release of iRsp1095 (see Methods). An initial refinement process resulted in addition of 280 reactions, 81 metabolites and 27 genes to iRsp1095, while removing 23 reactions and 3 genes (Additional file 3: Tables S1–3). Ninety-six of these 280 additional reactions consisted of newly added enzymatic activities, for which no apparent genes were present in the annotated *R. sphaeroides* genome. To identify candidate enzymes for these reactions, we conducted new BLAST analyses utilizing protein sequence of experimentally verified enzymes from other organisms. These searches resulted in the identification and annotation of 6 new candidate enzymes. An additional 11 enzymes already included in iRsp1095, with previously predicted functions, were identified as candidates to catalyze new reactions in the model (Additional file 3: Tables S4).

Of the 280 reactions added to iRsp1095, 92 are predicted membrane transport reactions. Organisms utilize a variety of transporters to import nutrients [37,38]. However, *R. sphaeroides* appears to favor the use of ATP-dependent ABC transporters (~7.84% of its genome is dedicated to ABC transport functions compared to 4.9% in *E. coli*[39]). An analysis of the *R. sphaeroides* genome reveals that of the genes encoding ABC transporters with no known or predicted functions, 11 are organized in operons encoding proteins with an ATPase domain, an integral membrane permease domain and a substrate-specific substrate binding domain (typical of importers [40]) (Table 4). To test the function of these putative transport operons, we deleted the gene for each of these 11 predicted substrate binding proteins and assessed growth of the resulting mutants using PM. This analysis, plus subsequent liquid growth studies led to identification of substrate-specific growth defects in mutants lacking individual genes for candidate transporters (Additional file 2: Figure S6). Based on this data set we have provisionally identified substrates for 5 previously uncharacterized transporters (Table 4). These transport reactions were incorporated into iRsp1095 by the addition of 15 genes, 2 reactions and one metabolite (Additional file 3: Tables S4).

**Table 4:** **Predicted *R. sphaeroides *ABC transporter operons tested for substrate specificity using Biolog PM**

**Operons**	**Identified substrate**^**a**^
RSP_0200-1, RSP_6101	Ribitol
RSP_0342-5	Methyl D-lactate
RSP_0346-9	Asparagine
RSP_1442-5	D-serine
RSP_2208-11	Uridine
RSP_0644-6	ND
RSP_2596-8	ND
RSP_2809-11	ND
RSP_3166-8	ND
RSP_3500-3	ND
RSP_3557-60	ND

^a^ND - Not Determined. Specific substrates for these transporters could not be determined from PM assay and the analysis of mutants lacking single genes in each of these predicted operons (see text).

These updates resulted in a refined metabolic model for *R. sphaeroides*, iRsp1140, consisting of 1416 reactions, 878 metabolites and accounting for 1140 genes within its genome (other properties of iRsp1140 are summarized in Table 5). Overall, iRsp1140 includes a larger number of reactions, metabolites and genes, and it is supported by increased physiological, genetic and genomic evidence for the inclusion of many components in the network. The predictions of iRsp1140 are in better agreement with the PM data compared with those of iRsp1095 (Figure 7), verifying the improved predictive ability of iRsp1140. In addition, predictions made by iRsp1140 for growth rate or production of H_2_, polyhydroxybutyrate and CO_2_ evolution were in excellent agreement with published data [11] (Additional file 3: Table S5). We expect that iRsp1140 will enable improved modeling of metabolic behavior under a larger number of conditions. In addition, the increased number of genes in iRsp1140 provides a larger set of targets for assessing the effects of genetic perturbations on growth or metabolic activity under both photosynthetic and non-photosynthetic conditions.

**Table 5:** Comparison of the properties of iRsp1095 and iRsp1140

**Categories**	**iRsp1095**	**iRsp1140**
**Genes**	**1095**	**1140**
Genes based on experimental evidence	334 (30.5%)	354 (31%)
Genes inferred based on gene homology	761 (69.5%)	786 (69%)
**Metabolites**	**796**	**878**
**Reactions**	**1158**	**1416**
Enzymatic Reactions	858	953
Transport reactions	300	463
Reactions Associated with genes	1049	1204
Spontaneous/Diffusion reactions	14	17
Reactions without gene association	95	195
Reversible Reactions	757	942
Irreversible Reactions	401	474
Exchange Reactions	148	231
Demand Reactions	3	3

## Conclusions

While the development of high-throughput or global approaches can provide large amounts of data, the task of extracting meaningful biological insight from this information is still challenging [4]. To gain new biological insights, constraint-based and other modeling approaches can be used to integrate various data sets [1-3,41].

In this study, we took an integrated approach to gain new insight into the metabolic, energetic and photosynthetic lifestyles of *R. sphaeroides*. We extended the number of nutrients that can support growth of WT cells. We also showed that a conserved bioenergetic enzyme (PntAB) which can provide reduced pyridine nucleotides is essential for photosynthetic growth on many carbon sources. We used a genome-scale model for *R. sphaeroides* to make flux predictions, as well as generate and test hypotheses on alterative NADPH-generating pathways that allow growth in the absence of PntAB. The products of various anabolic pathways require NADPH that is derived mainly from PntAB activity, so exploiting these and other alternative NADPH generating pathways we identified, could improve growth and metabolic end products derived from photosynthetic and non-photosynthetic wild type cells or those engineered to produce compounds of biotechnological interest.

Previous studies have shown the utility of high-throughput data sets in refining and validating genome-scale metabolic models [42-44]. We used our data to produce a 2^nd^ generation genome-scale reconstruction for *R. sphaeroides*, iRsp1140 with significantly improved coverage of metabolic functions and predictions that are in better agreement with experimental data. iRsp1140, provides an improved depiction of the *R. sphaeroides* metabolic network, so it will be useful in studying photosynthesis, as well as a wider range of metabolic processes in this and related organisms.

## Methods

### Bacterial strains and growth conditions

*R. sphaeroides* strains 2.4.1 and Ga were used as parental strains. All mutants were made in strain 2.4.1 except PntA1 [31] and Zwf1, which were constructed in Ga, and PntA1-Zwf1, which was constructed in PntA1 (Additional file 4: Table S1). *E. coli* DH5α was used as a plasmid host, and *E. coli* S17-1 was used to conjugate DNA into *R. sphaeroides*.

*R. sphaeroides* cultures were incubated at 30°C in Sistrom’s Minimal Medium (SMM) [45] lacking glutamate and aspartate, with succinate (33.9 mM), or an alternative sole carbon source. The molar concentration of carbon atoms of the carbon source was kept constant at 135.5 mM. Photosynthetic cultures were incubated in screw capped tubes at a light intensity of ~10 W/m^2^, while anaerobic respiratory cultures were incubated in screw capped tubes in the dark with the media supplemented with 0.9% DMSO. Aerobic cultures were shaken in flasks. Optical density of photosynthetic cells in screw capped tubes was measured using a Klett-Summerson photometer and is expressed in Klett units (1 Klett unit equals approximately 10^7^ cells/mL). Other optical density measurements were made by measuring optical density at 600 or 650 nm in a spectrophotometer. When required, the media was supplemented with 100 μM IPTG, 25 μg/mL kanamycin or 25 μg/mL spectinomycin. *E. coli* cells were grown in Luria Bertani medium at 37°C, supplemented with 25 μg/mL kanamycin where needed.

### Phenotype microarray analysis

To determine the substrate utilization profiles of *R. sphaeroides*, phenotype microarrays (PMs) were used with a few modifications. To assay aerobic respiratory growth on different carbon sources (Biolog PM1 and PM2A), cells were grown on SMM agar plates aerobically for 3 days. Cells were swabbed from the agar plates and suspended in 4 mL inoculation fluid (Biolog inc.) to an OD_600_ of 0.38. Two mL of this mixture was placed in 10 mL of inoculation fluid (IF) containing 24 μL of tetrazolium-based dye A (Biolog inc.), resulting in a final OD_600_ of ~0.07. Then 1.2 μL of vitamin solution (1 g nicotinic acid, 0.5 g thiamine-HCl and 0.01 g biotin in 100 mL of water) was added and 100 μL was dispensed into each well of a 96 well plate. Cultures were incubated at 30˚C for 72 to 96 hrs in an OmniLog plate reader (Biolog inc).

To assay photosynthetic growth, 10 mM NaHCO_3_, 0.4 mM sodium thioglycolate and 1 μM methylene green were added to the IF and this was kept in an anaerobic chamber for ~7 days with periodic shaking to facilitate it becoming anaerobic (the methylene green in the IF turns colorless once oxygen is removed). *R. sphaeroides* cells were grown photosynthetically on SMM agar plates and PM plates set up as described above, except that the tetrazolium dye was omitted, as thioglycolate reduces the tetrazolium-based dye turning it purple independent of cellular respiration. The PM plates were put in an anaerobic chamber, inoculated, placed in heat sealed anaerobic bags (Biolog inc.) [46] and incubated under constant illumination (light intensity of ~10 W/m^2^) at 30˚C for 72 to 96 hrs, after which OD_650_ readings were taken. Anaerobic indicator strips and ageless oxygen absorbers (MITSUBISHI Gas Chemical America, Inc.) were placed in the sealed bags to report on and maintain an anaerobic environment throughout the experiment.

Any growth in the negative control well (A1) was subtracted from the measured optical density for both aerobic and photosynthetic PM. A threshold OD_650_ of 0.05 (after background correction) was used as a baseline for scoring photosynthetic growth, as this was the highest value obtained from any well in control experiments where cells were kept in the dark. A threshold of 5 Omnilog units (after background correction) was used as a baseline for aerobic respiratory growth. Only carbon sources that resulted in reproducible growth above the baseline across all replicates were considered to be growth substrates.

To assay photosynthetic growth on different nitrogen, phosphorus or sulfur sources (Biolog PM3B and PM4A), *R. sphaeroides* cells were grown aerobically for 5 days on a modified R2A agar [22,47] (0.25 g of yeast extract, 0.25 g of Proteose Peptone, 0.25 g of Casamino Acids, 0.12 g of K_2_HPO_4_, 0.025 g of MgSO_4_.7H_2_O, 0.5 g of sodium pyruvate and 15 g of agar per liter of water). Plates were set up as described for photosynthetic growth with the addition of 20 mM sodium succinate and 2 μM ferric citrate. Cell cultures were grown under constant illumination (10 W/m^2^) at 30˚C for 48 hrs, after which OD_650_ readings were taken. Aerobic growth on the different nitrogen, phosphorus and sulfur sources is not reported due to significant background growth in the negative control wells, an issue that has been observed previously [25].

Due to comparatively slow growth rates of *R. sphaeroides* under anaerobic respiratory conditions, evaluation of these growth modes could not be reliably conducted with Biolog PM plates due to media evaporation. Thus, to assay anaerobic respiratory growth on different carbon sources, 96 well microwell plates (Fisher Scientific) were set up to analyze 41 of the carbon sources identified as *R. sphaeroides* growth substrates from PM (see Additional file 1: Table S1 for a list of these substrates). The carbon sources were normalized for the total number of carbon atoms in each compound (135.5 mM). *R. sphaeroides* cells were grown aerobically in SMM and centrifuged. Cells were then washed with SMM media lacking a carbon source (SMM no carbon), suspended in anaerobic SMM no carbon (which had been kept in an anaerobic chamber for at least 4 days) to an OD_600_ of ~0.1 and DMSO was added to a final concentration of 0.9%. Then, 300 μL of a cell suspension was dispensed into wells containing a different carbon source in an anaerobic chamber. Plates were incubated at 30˚C for 10 days with continuous shaking in a Tecan M200 plate reader located within the anaerobic chamber, with OD_650_ readings taken every 6 minutes. Alternatively, plates were sealed in anaerobic bags, wrapped in foil and incubated at 30˚C for 10 days with periodic shaking.

### Construction of mutants

All *R. sphaeroides* mutants we constructed contained in-frame markerless deletions. Briefly, regions spanning ~1500 bp upstream and downstream of the target gene were amplified using sequence specific primers containing restriction sites for EcoRI, XbaI or HindIII. These fragments were digested with the appropriate restriction enzymes and ligated into pK18mobsacB plasmid [48], digested with EcoRI and HindIII, by three-way ligation to generate the various gene deletion constructs, which were confirmed by sequencing (Additional file 4: Table S1 and S2). The pK18mobsacB-based plasmids were separately mobilized from *E. coli* S17-1 into *R. sphaeroides* strains. Cells in which the plasmid had successfully integrated into the genome via homologous recombination were identified by selection on SMM plates supplemented with kanamycin. These cells were then grown overnight in SMM without kanamycin. Cells that had lost the deletion plasmid (and thus the *sacB* gene) via a second recombination event were identified by growth on SMM plates supplemented with 10% sucrose. Individual gene deletions were confirmed by PCR and sequencing with specific primers.

Ectopic expression plasmids were made by amplifying the target genes from the genome using sequence specific primers containing restriction sites for NdeI and BglII, HindIII or BamHI. These DNA fragments were digested with the appropriate enzymes and cloned into pIND5 digested with the same enzymes. These plasmids were conjugated from *E. coli* S17-1 into the relevant *R. sphaeroides* mutant. Cells which harbor the desired plasmid were identified by selection on SMM plates supplemented with kanamycin.

### RNA extraction, qRT-PCR and microarray analyses

RNA was isolated from exponential phase cultures of *R. sphaeroides* strains that were grown either photosynthetically in 16 mL screw cap tubes or aerobically in 500 mL conical flasks. RNA isolation and subsequent cDNA synthesis were performed as previously described [49]. qRT-PCR experiments were conducted in triplicate for each biological replicate using SYBR Green JumpStart Taq ReadyMix (Sigma-Aldrich). Relative expression was determined via the 2-^ΔΔC^_T_ method with efficiency correction [50]. *R. sphaeroides rpoZ* was used as a housekeeping gene for normalization. Primers used in this analysis are provided in Additional file 4: Table S2.

### Constraint based analysis and model refinement

Separate stoichiometric matrices, S_m x n_, were generated from the reconstructions (i.e., iRsp1095 and iRsp1140) with the rows (m) representing metabolites, the columns (n) representing reactions and entries in the matrices representing stoichiometric coefficients for metabolites involved in each reaction. Flux balance analysis (FBA) [51] was used to simulate *in silico* growth by solving the linear programming problem:

$$\begin{array}{l} \max\;v_{\mathrm{Biomass}}\\ s.t\\ S•v=0\\ v_{\min}\leq v\leq v_{\max} \end{array}$$

where v_Biomass_ is the flux through biomass objective function; **v** is the vector of steady state reaction fluxes; and **v**_min_ and **v**_max_ are the minimum and maximum allowable fluxes. The values in **v**_min_ and **v**_max_ were set to −100 and 100 mmol/g DW h for reversible reactions and 0 and 100 mmol/g DW h for forward only reactions. During simulation, all exchange reactions were assigned as being forward only (allowing metabolites to be secreted into the medium but not taken up), except the exchange reactions for media components required for cell growth, which were set to measured values for limiting substrates such as ammonia, or allowed to be freely exchanged with the extracellular space, i.e., -100 ≤ v ≤ 100. The non-growth associated ATP maintenance limit was set to 8.39 mmol/gDW h [24]. Flux variability analysis [52] was also used to determine minimum and maximum allowable flux through reactions in the network.

Initial simulations with iRsp1095 in which the transhydrogenase reaction was deleted resulted in the prediction of optimal growth, suggesting alternative NADPH generating reactions existed in the metabolic network. Analysis of iRsp1095 revealed that it includes at total of 61 NADPH requiring reactions, of which only 29 were independently non-essential and capable of functioning in the direction of NADPH synthesis. To identify all reactions within iRsp1095 capable of producing NADPH to support growth, all 29 non-essential NADPH-requiring reactions within iRsp1095, capable carrying flux the direction of NADPH synthesis, were turned off. This resulted in a predicted growth rate of 0. Optimal growth was restored solely by turning on the transhydrogenase reaction, consistent with transhydrogenase being sufficient for generating NADPH required for growth. All other reactions capable of independently restoring growth, while the other NADPH-requiring reactions were still off, were considered as a candidate NADPH producing reaction (Table 3).

To assess the predicted NADPH demand during aerobic or photosynthetic growth across growth substrates (i.e., succinate, glucose and acetate), all predicted NADPH generating reactions (Table 3) set to have a zero flux, except the transhydrogenase reaction. Using a previously described mixed integer linear programming approach [53,54], 1000 alternative optimal FBA solutions were identified under each condition. The flux through the transhydrogenase reaction, and thus the amount of NADPH predicted to be required, under each condition was averaged over the 1000 alternative optimal solutions and this average was used as an estimate of NADPH demand under those conditions.

To predict fluxes through central metabolism, we used an extension of FBA called E-flux [55]. E-flux limits the maximum and minimum fluxes (**v**_max_ and **v**_min_ respectively) through the reactions in the network based on genome-wide gene expression measurements. To achieve this publicly available microarray data obtained from cells grown on succinate and glucose (GEO platform GPL162), as well as from cells grown acetate (this study), were normalized and used to constrain the fluxes through each reaction in the network as previously described [55]. For reactions without gene-to-protein-to-reaction (GPR) assignments, the fluxes through these reactions were allowed to have a **v**_max_ of 100 mmol/g DW h and a **v**_min_ of 0 or −100 mmol/g DW h, if the reactions were forward only or reversible respectively. For reactions catalyzed by isozymes, the expression value of gene for the isozyme with the highest expression was used to constrain the reaction, while for multi-subunit enzymes the gene for the subunit with the lowest expression was used to constrain the reaction. After setting the upper and lower bounds, subsequent simulations were conducted with FBA as described above.

The previously published genome-scale model for *R. sphaeroides* iRsp1095 [11] was used as the starting point for a 2-step model refinement. In the first step, PM data was used to guide model refinement, which involved the manual addition and removal of reactions to bring it into better alignment with the PM data. PM data for carbon (C), nitrogen (N), phosphorus (N) and sulfur (S) utilization were compared to model predictions from FBA simulations in which equivalent compounds were provided as the sole sources of these nutrients. The uptake rate of the tested carbon source was set to −4 mmol/g DW h, while that of N, P or S sources was set to −1 mmol/g DW h, as these are in the range of uptake rates typically observed in *R. sphaeroides*[11]. Succinate was used as the C source when testing for N, P and S utilization, while ammonium, inorganic phosphate and inorganic sulfate served as N, P and S sources when accessing C utilization (consistent with the PM analysis). For these simulations, nutrients which resulted in a FBA predicted growth rate greater than zero were considered growth substrates. **N**o **G**rowth-**G**rowth (NGG – no growth predicted by model but growth observed in PM) inconsistencies were manually rectified by addition of appropriate transport and/or enzymatic reactions from the multi-organism databases KEGG [56] and BRENDA [57,58]. The required enzymatic reaction(s) were added to the model based on one of the following 2 criteria: (i) the presence genes in the *R. sphaeroides* genome encoding the proteins potentially capable of catalyzing the new reaction(s) to be added; and (ii) if no putative enzymes were identified, the metabolic route that required the addition of the fewest reactions to iRsp1095 was selected. **G**rowth-**N**o **G**rowth (GNG – growth predicted by model but no growth observed in PM) inconsistencies in iRsp1095 were resolved by removal of transport reactions for the substrate in question, when this did not introduce and new inconsistencies with the PM data.

In a second step of model refinement, putative enzymes capable of catalyzing reactions added to iRsp1095 where identified by BLAST searches using the protein sequences from other organisms previously verified to carry out the reaction in question. A BLAST E-value cutoff of 10e-^20^ was selected as a threshold for significance. Enzymatic functions not previously included in iRsp1095 and which were encoded by genes without any previously defined specific function were considered as newly annotated genes, while those with previously defined putative functions were considered as having additional functionality (Additional file 3: Table S4). Updated information from KEGG [56] database, new information obtained from mutant analysis in this study, and data from recent literature searches were incorporated to generate iRsp1140. iRsp1140 in SBML format is provided in Additional file 5 and can be accessed in the BioModels database with ID MODEL1304240000.

## Abbreviations

DMSO: Dimethyl sulfoxide; PM: Phenotype microarray; FBA: Flux balance analysis; NADPH: Nicotinamide adenine dinucleotide phosphate; NADH: Nicotinamide adenine dinucleotide.

## Competing interests

The authors declare that they have no competing interests.

## Authors’ contributions

SI participated in reconstruction, curation and assessment of iRsp1140 and performed all the experiments in this paper. TJD and DRN conceived of project and coordinated research. SI wrote paper with critical reading and revisions by DRN and TJD. All authors contributed to and approved the contents of the final manuscript.

## Supplementary Material

Additional file 1 **Phenotype microarray data.** This is an excel file containing results from Biolog phenotype microarray analysis of *R. sphaeroides* wild-type and mutant cells across a variety of growth conditions and nutrient sources. It contains eight tables (Tables S1-8). Click here for file

Additional file 2 **Additional figures.** File containing additional figures S1-S6. Click here for file

Additional file 3 **Details of iRsp1140.** Excel file containing tables with the details of the metabolic model iRsp1140. It contains eight tables (Tables S1-8). Click here for file

Additional file 4 **Strains and Plasmids.** An excel file with strains and plasmids used in this paper. It contains 2 tables (Tables S1 and S2). Click here for file

Additional file 5 **iRsp1140 in SBML format.** SBML format of iRsp1140 for distribution and use in other modeling environments. Click here for file
