# Supplementary material for: Global insights into energetic and metabolic networks in Rhodobacter sphaeroides
Source: BMC Syst Biol. 2013 Sep 13;7:89. doi: 10.1186/1752-0509-7-89 (PMC3849096; doi:10.1186/1752-0509-7-89)
Supplement: Additional file 2 — Additional figures. File containing additional figures S1-S6. [file 1752-0509-7-89-S2.pdf]

Additional Figure Legends

Figure S1. *R. sphaeroides* growth phenotypes under different conditions. (A) Venn diagram depicting the overlap in carbon utilization between aerobic respiratory and photosynthetic growth conditions based on PMs, and their overlap with predictions from the genome-scale metabolic model for *R. sphaeroides*, iRsp1095. The 32 carbon sources predicted to be used only by iRsp1095 included 11 compounds not included on Biolog PMs and 21 false positive predictions. (B) Curve showing *R. sphaeroides* growth on inosine under aerobic (○) and photosynthetic conditions (□). During aerobic respiratory growth there is a lag period (>72hrs). The red triangle indicates the time at which PM analyses were typically ended. Thus, the growth curve is consistent with the PM data. Additionally, stationary phase is reached at much lower optical density under aerobic respiratory than photosynthetic conditions (74 compared to 296 Klett units, respectively) (C) Curve showing *R. sphaeroides* growth on isoleucine under aerobic respiratory (○) and photosynthetic conditions (□). No significant growth is observed photosynthetically using isoleucine as a carbon source after extended incubation.

Figure S2. Growth of *R. sphaeroides* strains on agar plates under different conditions. *R. sphaeroides* strains (WT, PntA1, PntA1+PntAB, Zwf1, Zwf1+Zwf and PntA1-Zwf1) grown on agar plates under aerobic and photosynthetic conditions on (A) glucose, (B) acetate and (C) succinate as a sole carbon source. PntA1 is incapable of photosynthetic growth on either acetate or succinate. Zwf1 showed severely impaired growth on glucose both aerobically and photosynthetically, indicating it is essential for glucose metabolism in *R. sphaeroides* (note that while Zwf1 eventually grows photosynthetically on glucose agar plates, this growth is significantly impaired; Figure 5).

Figure S3: Predicted flux distributions during photosynthetic growth on succinate. Map of *R. sphaeroides* central carbon metabolism showing flux distributions predicted by iRsp1095 during photosynthetic growth with succinate. All fluxes are in mmol/gDW h. The fluxes in black are those predicted for the wild type cells, while those in red are predictions made for the Δ*pntAB* deletion strain. Reactions with no flux values are predicted to have a zero flux. To improve accuracy of predictions, fluxes were constrained using publicly available genome-wide expression data from wild type cells grown photosynthetically on succinate (see Materials and Methods). Green arrows indicate the predicted NADPH generating reactions under these conditions.

Figure S4: Predicted flux distributions during photosynthetic growth on glucose. Map of *R. sphaeroides* central carbon metabolism showing flux distributions predicted by iRsp1095 during photosynthetic growth with glucose. All fluxes are in mmol/gDW h. The fluxes in black are those predicted for the wild type cells, while those in red are predictions made for the Δ*pntAB* deletion strain. Reactions with no flux values are predicted to have a zero flux. To improve accuracy of predictions, fluxes were constrained using publicly available genome-wide

expression data from wild type cells grown photosynthetically on glucose (see Materials and Methods). Green arrows indicate the predicted NADPH generating reactions under these conditions.

Figure S5: Predicted flux distributions during photosynthetic growth on acetate. Map of *R. sphaeroides* central carbon metabolism showing flux distributions predicted by iRsp1095 during photosynthetic growth with acetate. All fluxes are in mmol/gDW h. Reactions with no flux values are predicted to have a zero flux. To improve accuracy of predictions, fluxes were constrained using gene expression data from wild type cells grown photosynthetically on acetate generated in this study (see Materials and Methods). Green arrows indicate the predicted NADPH generating reactions under these conditions.

Figure S6: Photosynthetic growth curves of substrate binding protein deletion mutants. Growth curves highlighting impaired growth phenotypes on specific carbon sources of 5 substrate binding protein deletion mutants. [a]Percentage represents agreement between predictions and PM data from photosynthetic analysis only. [b]The *ΔRSP_0345* mutant eventually grows on methyl D-lactate at a much slower rate (double time of ~15 hrs compared to 6.3 hrs in the WT), possibly due to an alternative less specific transporter.

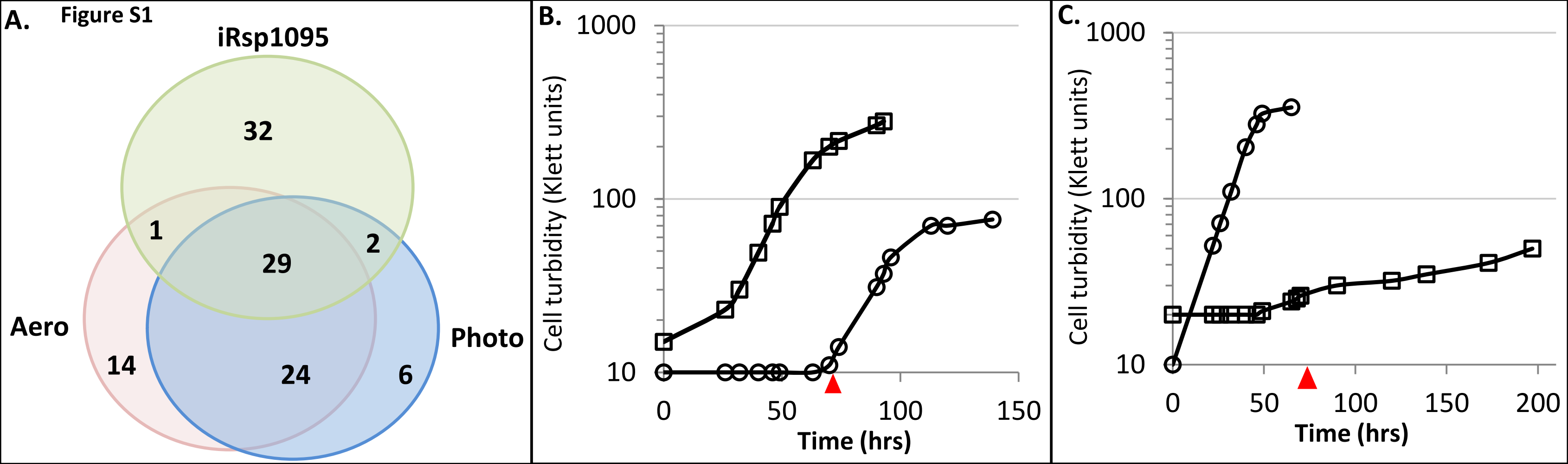
A.
Figure S1
iRsp1095
32
1
29
2
Aero
14
24
6
Photo
B.
1000
100
10
0
50
100
150
Cell turbidity (Klett units)
Time (hrs)
C.
1000
100
10
0
50
100
150
200
Cell turbidity (Klett units)
Time (hrs)

Figure S2

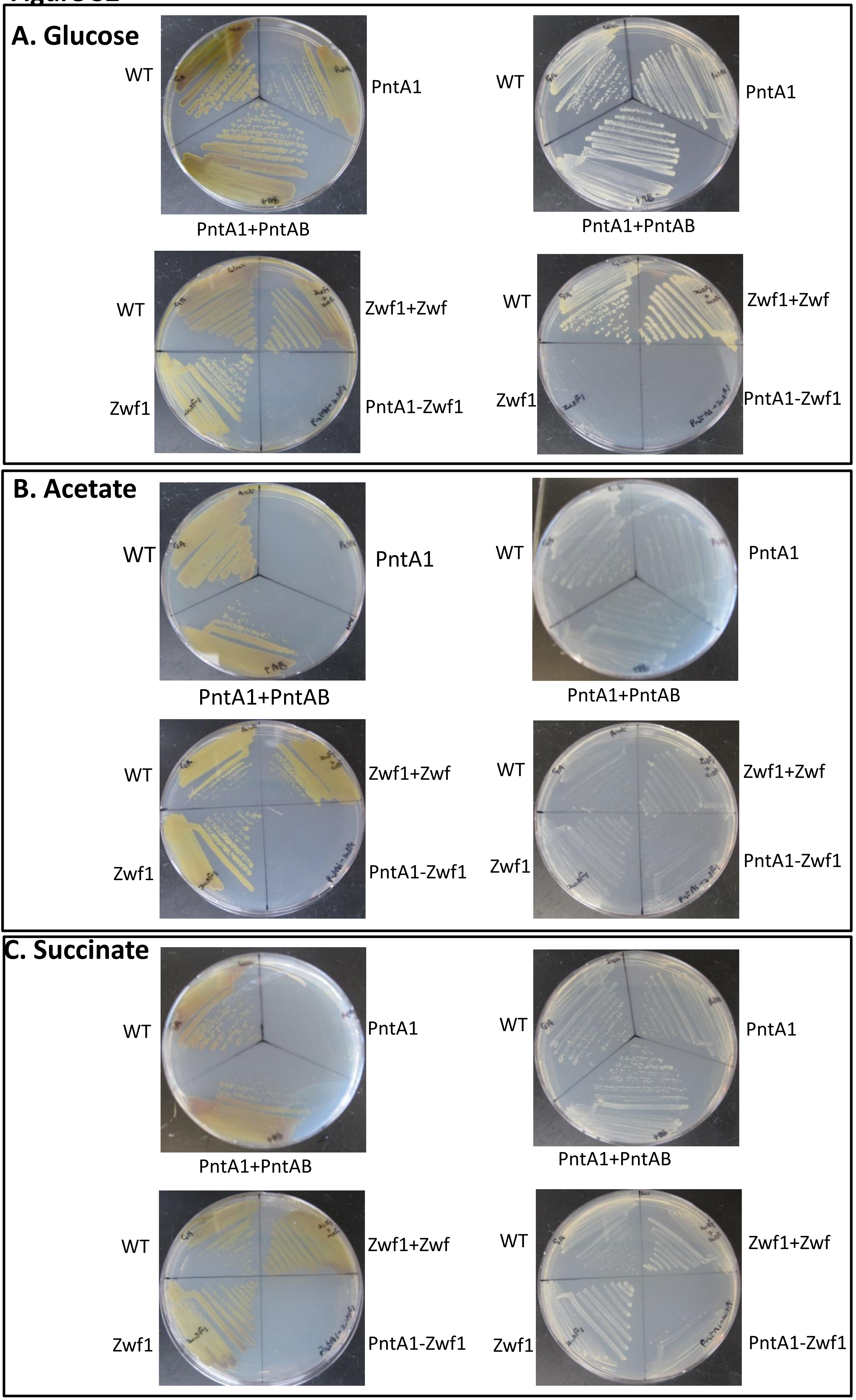
PHOTO
AERO
A. Glucose
WT
PntA1
PntA1+PntAB
WT
PntA1
PntA1+PntAB
WT
Zwf1+Zwf
Zwf1
PntA1-Zwf1
WT
Zwf1+Zwf
Zwf1
PntA1-Zwf1
B. Acetate
WT
PntA1
PntA1+PntAB
WT
PntA1
PntA1+PntAB
WT
Zwf1+Zwf
Zwf1
PntA1-Zwf1
WT
Zwf1+Zwf
Zwf1
PntA1-Zwf1
C. Succinate
WT
PntA1
PntA1+PntAB
WT
PntA1
PntA1+PntAB
WT
Zwf1+Zwf
Zwf1
PntA1-Zwf1
WT
Zwf1+Zwf
Zwf1
PntA1-Zwf1

Figure S3

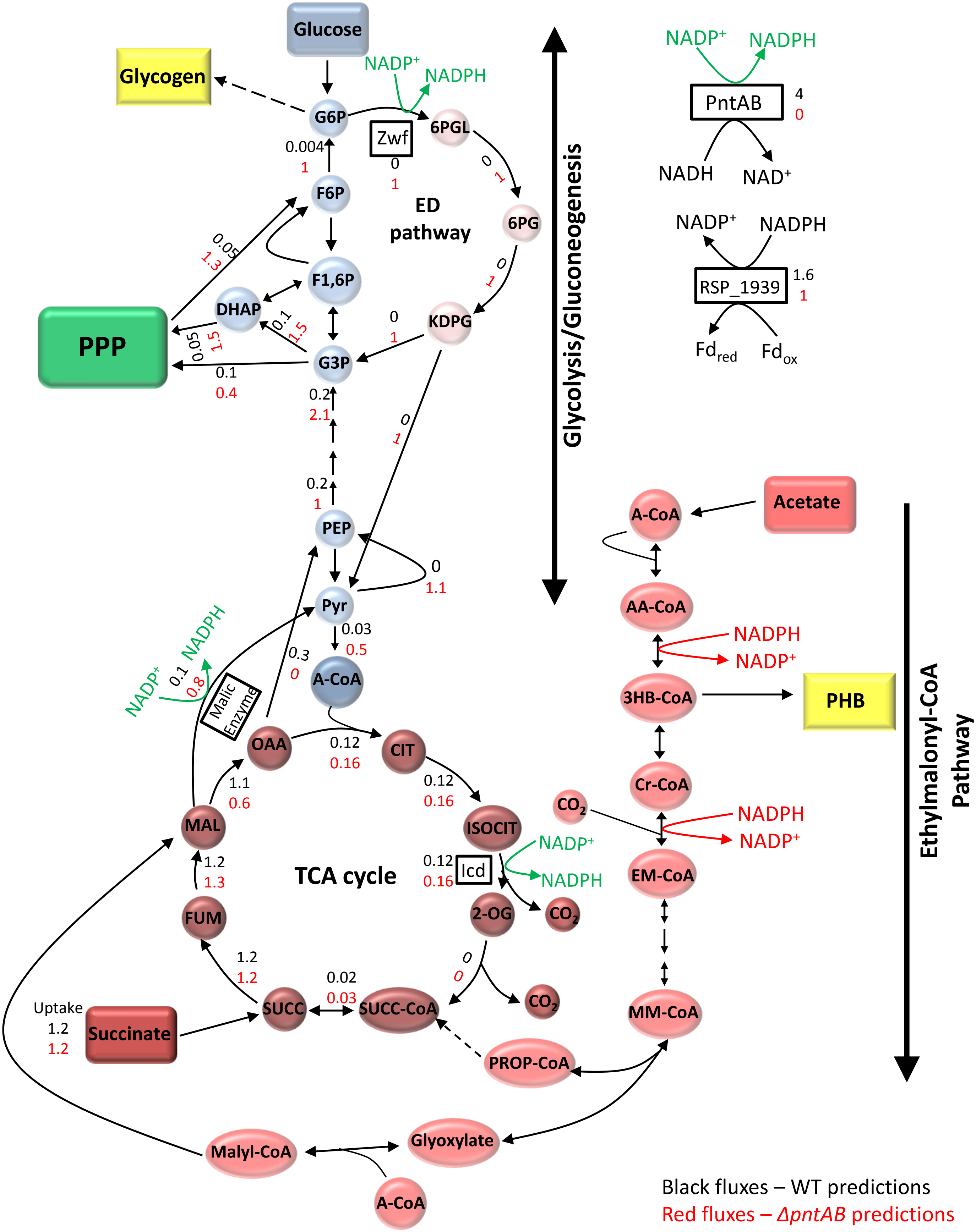

Glucose
Glycogen
NADP+
NADPH
G6P
6PGL
Zwf
0.004
1
0
1
F6P
ED
pathway
6PG
F1,6P
DHAP
PPP
G3P
KDPG
0.05
1.3
0.1
1.5
0.05
1.5
0.1
0.4
0.2
2.1
0.2
1
PEP
Pyr
0
1.1
0.03
0.5
A-CoA
Malic Enzyme
0.1
0.8
0.3
0
OAA
CIT
0.12
0.16
MAL
1.1
0.6
ISOCIT
Icd
NADP+
NADPH
CO2
2-OG
TCA cycle
FUM
1.2
1.3
SUCC
SUCC-CoA
0.02
0.03
Uptake
1.2
1.2
Succinate
PROP-CoA
Malyl-CoA
Glyoxylate
Glycolysis/Gluconeogenesis
PntAB
4
0
NADH
NAD+
RSP_1939
1.6
1
Fdred
Fdox
Acetate
AA-CoA
3HB-CoA
PHB
Cr-CoA
EM-CoA
MM-CoA
Ethylmalonyl-CoA Pathway
Black fluxes – WT predictions
Red fluxes – ΔpntAB predictions

Figure S4

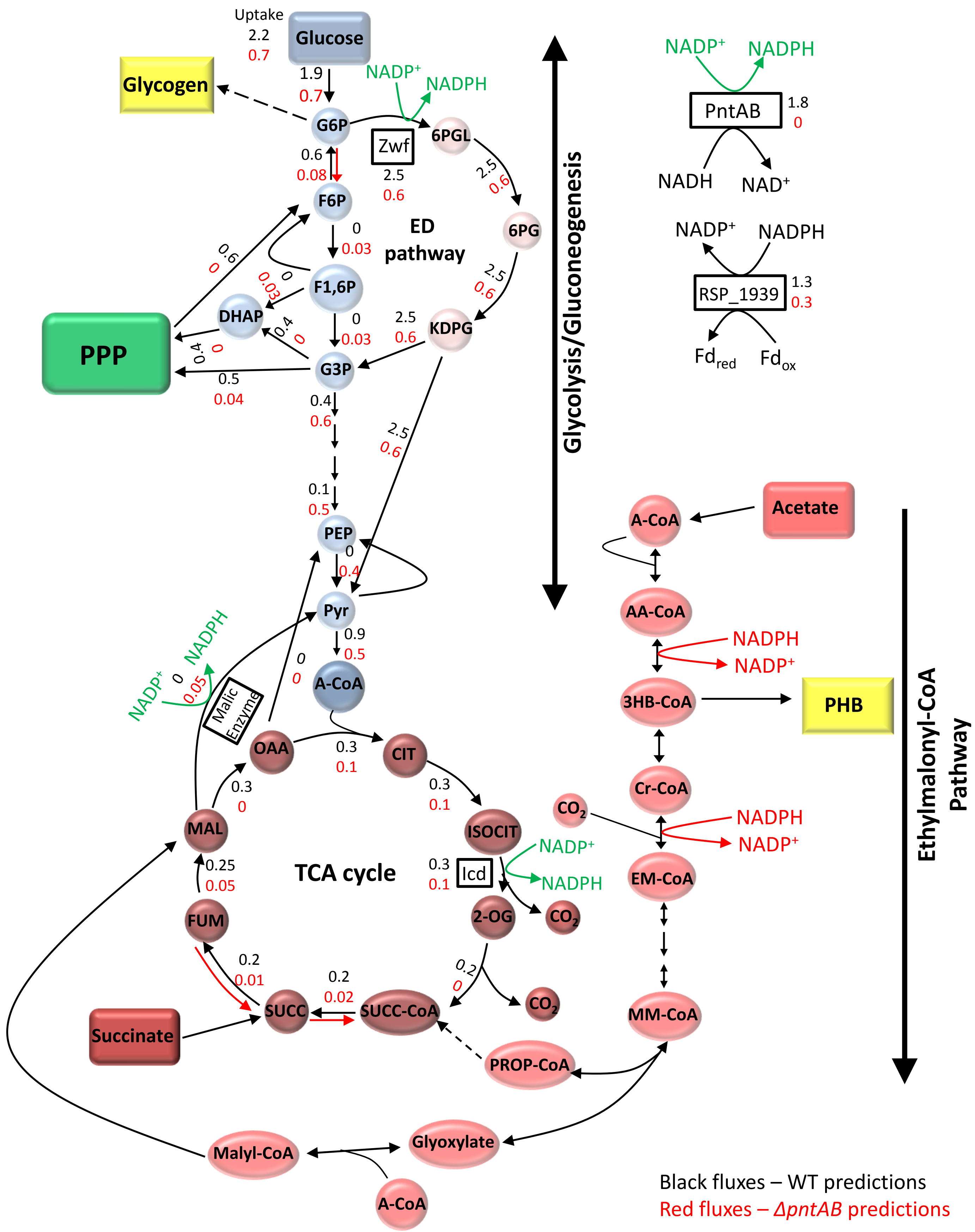

Uptake
2.2
0.7
Glucose
Glycogen
1.9
0.7
NADP+
NADPH
G6P
Zwf
6PGL
0.6
0.08
2.5
0.6
F6P
6PG
ED
pathway
0
0.03
F1,6P
DHAP
PPP
KDPG
G3P
0.5
0.04
0.4
0.6
0.1
0.5
PEP
0
0.4
Pyr
0.9
0.5
A-CoA
Malic Enzyme
OAA
CIT
0.3
0.1
MAL
ISOCIT
TCA cycle
Icd
NADP+
NADPH
0.25
0.05
FUM
2-OG
CO_2
0.2
0.01
0.2
0.02
SUCC
SUCC-CoA
Succinate
PROP-CoA
Malyl-CoA
Glyoxylate
A-CoA
Glycolysis/Gluconeogenesis
PntAB
1.8
0
NADH
NAD+
RSP_1939
1.3
0.3
Fd_{red}
Fd_{ox}
Acetate
AA-CoA
3HB-CoA
PHB
Cr-CoA
EM-CoA
MM-CoA
Ethylmalonyl-CoA Pathway
Black fluxes – WT predictions
Red fluxes – *ΔpntAB* predictions

Figure S5

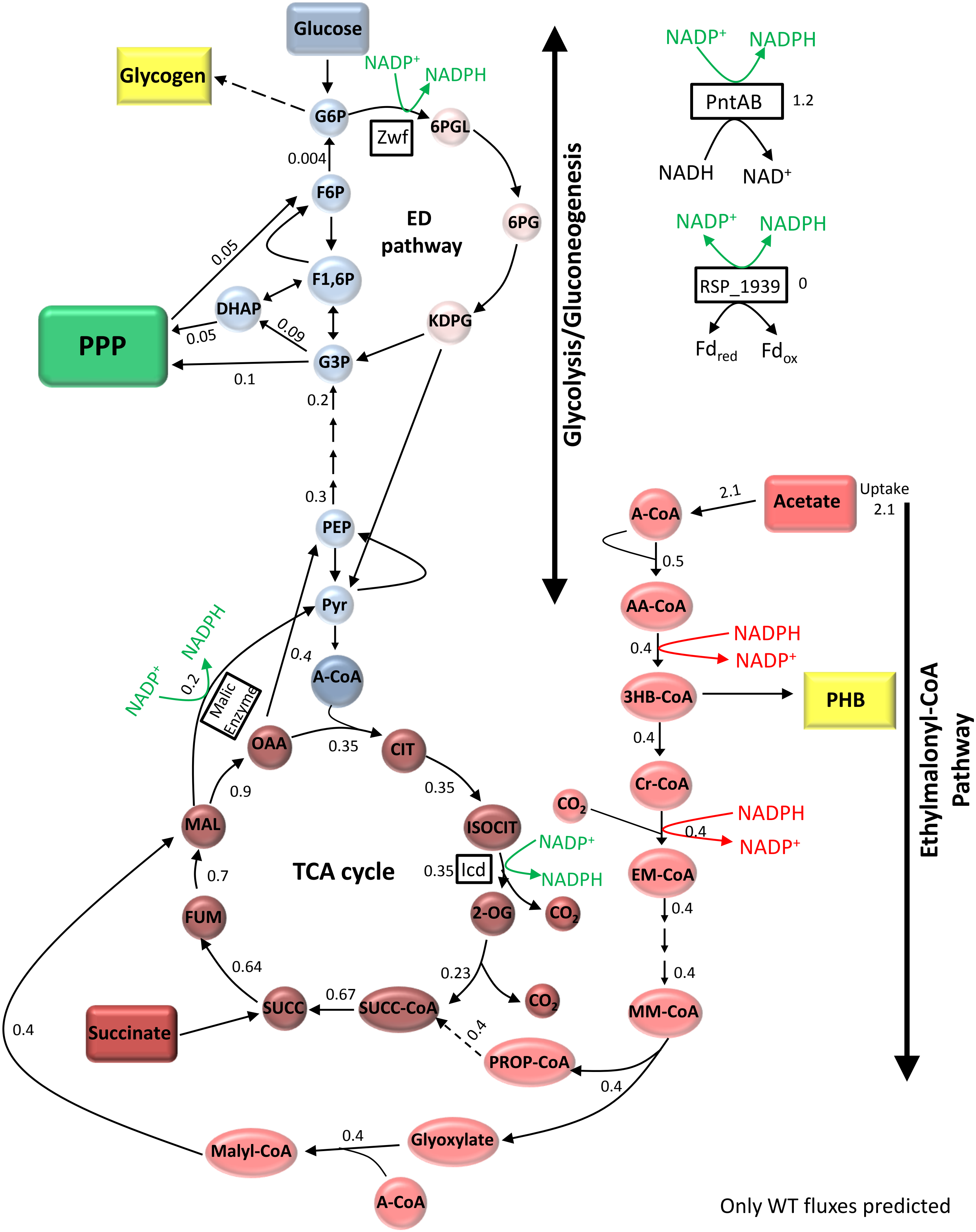

Only WT fluxes predicted

Figure S6

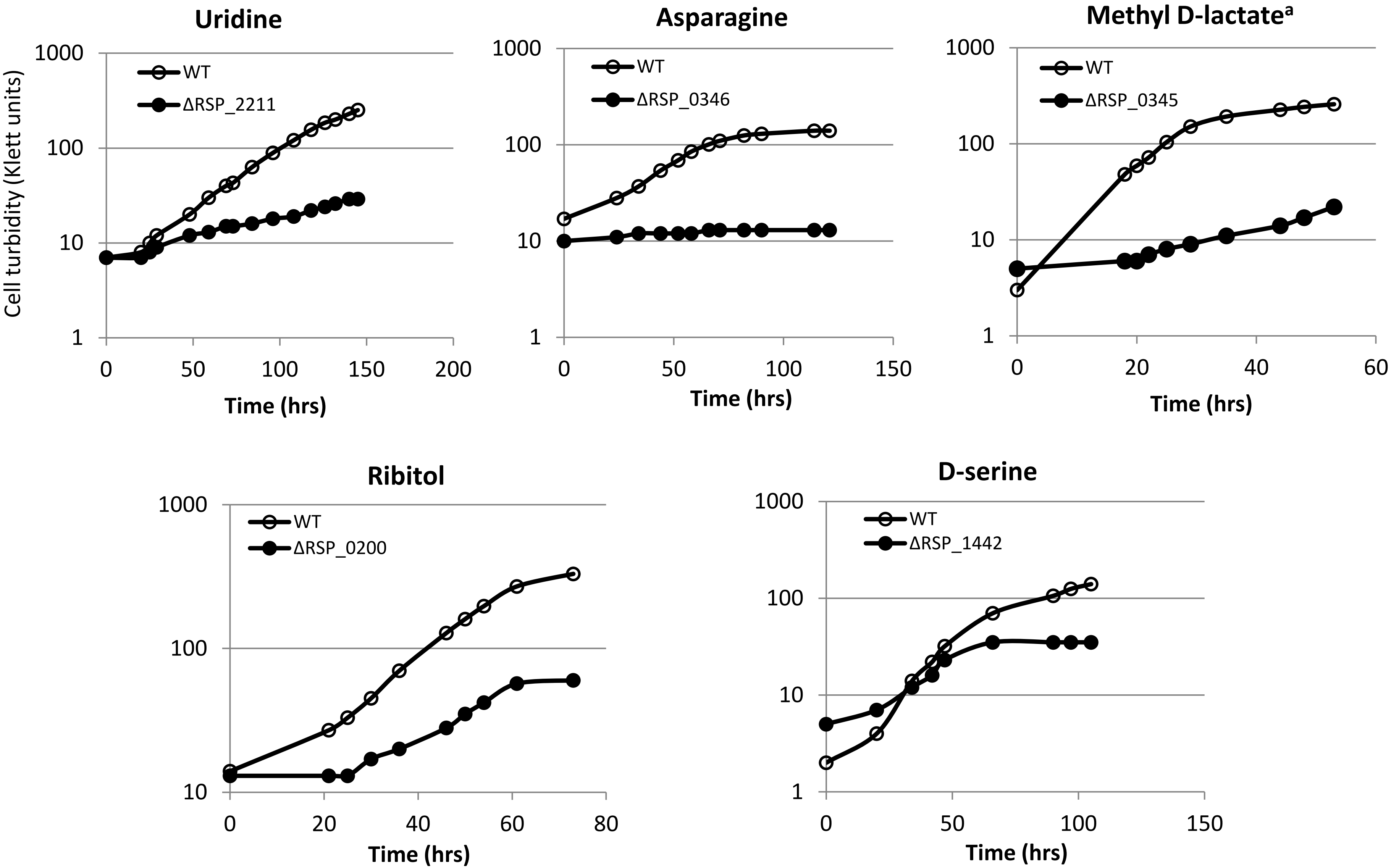

[a]The *ΔRSP_0345* mutant eventually grows on methyl D-lactate at a much slower rate (double time of ~15 hrs compared to 6.3 hrs in the WT), possibly due to an alternative less specific transporter.
